# Supplementary material for: Reconciling Mining with the Conservation of Cave Biodiversity: A Quantitative Baseline to Help Establish Conservation Priorities
Source: PLoS One. 2016 Dec 20;11(12):e0168348. doi: 10.1371/journal.pone.0168348 (PMC5173368; doi:10.1371/journal.pone.0168348)
Supplement: S1 Dataset — (ZIP) [file pone.0168348.s002.zip › Taxa/Serra Norte/SN_2013/N5S/Lista N5S-73.pdf]

CAVIDADE N5S-0073

Classe	Ordem	Fam/Outros	Gên/Outros	Espécie	S-U	U-S	Zon.
Annelida	Oligochaeta			jovem		3	P
Arachnida	Acari	Laelapidae	*Cosmolaelaps*	sp.1		X	P
Arachnida	Amblypygi	Phrynidae	*Heterophrynus*	*longicornis*	3		P
Arachnida	Araneae	Ochyroceratidae	*Speocera*	sp.1		X	P
Arachnida	Araneae	Oonopidae	aff. *Xyccarph*	sp.3	X	x	P
Arachnida	Araneae	Pholcidae	*Mesabolivar*	*aurantiacus*	X	X	P
Arachnida	Araneae	Pholcidae	*Mesabolivar*	sp.1	X		P
Arachnida	Araneae	Pisauridae	*Architis*	sp.1	2		P
Arachnida	Araneae	Scytodidae	*Scytodes*	sp.1	1		P
Arachnida	Araneae	Theraphosidae		jovem	1		P
Arachnida	Araneae	Theridiidae	*Episinus*	sp.1	X		P
Arachnida	Araneae	Theridiidae	*Theridion*	sp.2	X		P
Arachnida	Araneae	Theridiosomatidae	*Plato*	sp.1	X	X	P
Arachnida	Araneae	Trechaleidae		jovem	1		P
Arachnida	Opiliones	Sclerosomatidae	*Prionostema*	sp.1	1		P
Arachnida	Pseudoscorpiones	Chthoniidae		jovem		X	P
Diplopoda	Polydesmida	Fuhrmannodesmidae		jovem		X	P
Entognatha	Collembola	Cyphoderidae		sp.1		X	P
Entognatha	Collembola	Isotomidae		sp.1		X	P
Insecta	Blattodea			jovem	1	1	P
Insecta	Coleoptera	Chrysomelidae		sp.8	X		P
Insecta	Coleoptera	Curculionidae	Scolytinae	sp.1		X	P
Insecta	Coleoptera	Dytiscidae		sp.5	X	X	P
Insecta	Coleoptera	Dytiscidae		sp.8	X	X	P
Insecta	Coleoptera	Dytiscidae		sp.9		X	P
Insecta	Coleoptera	Hydrophilidae		sp.2		X	P
Insecta	Coleoptera	Staphylinidae	Pselaphinae	sp.2	X		P
Insecta	Coleoptera	Staphylinidae	Pselaphinae	sp.4	X		P
Insecta	Coleoptera	Staphylinidae		sp.8		X	P
Insecta	Coleoptera	Staphylinidae		sp.9		X	P
Insecta	Coleoptera	Staphylinidae		sp.21	X		P
Insecta	Diptera	Chaoboridae		sp.	X		P
Insecta	Diptera	Psychodidae	Phlebotominae	sp.	X	x	P
Insecta	Diptera	Tipulidae	Tipulinae	sp.	X		P
Insecta	Heteroptera	Cydnidae		jovem		X	P
Insecta	Heteroptera	Dipsocoroidea		jovem		X	P
Insecta	Heteroptera	Naucoridae		sp.	X		P
Insecta	Heteroptera	Tingidae		jovem		X	P
Insecta	Heteroptera	Veliidae	*Paravelia*	sp.	X	X	P
Insecta	Hymenoptera	Formicidae	*Dolichoderus*	aff. *bispinosus*	X		P
Insecta	Hymenoptera	Formicidae	*Pachycondyla*	sp.2	X	X	P
Insecta	Hymenoptera	Formicidae	*Paratrechina*	sp.1	X		P
Insecta	Hymenoptera	Formicidae	*Pheidole*	sp.4	X		P
Insecta	Hymenoptera	Formicidae	*Solenopsis*	sp.1		X	P
Insecta	Isoptera	Termitidae	*Armitermes*	sp.		X	P
Insecta	Isoptera	Termitidae	*Nasutitermes*	sp.	X		P
Insecta	Lepidoptera	Noctuoidea		sp.6	3		P
Insecta	Orthoptera	Phalangopsidae	*Paraclodes*	sp.	5		P
Insecta	Orthoptera	Phalangopsidae	*Phalangopsis*	sp.	20	8	P

Malacostraca	Isopoda	Philosciidae		sp.1	X	X	P
Turbellaria				sp.4	X	X	P
Amphibia	Anura	Leptodactylidae	*Leptodactylus*	cf. *vastus*	3	3	P
Amphibia	Anura	Strabomantidae	*Pristimantis*	cf. *fenestratus*	2		P
